# Uncovering the Genetics and Physiology behind Optic Neuritis

**DOI:** 10.3390/genes14122192

**Published:** 2023-12-09

**Authors:** Ilaria Del Negro, Giada Pauletto, Lorenzo Verriello, Leopoldo Spadea, Carlo Salati, Tamara Ius, Marco Zeppieri

**Affiliations:** 1 Clinical Neurology Unit, Head-Neck and Neurosciences Department, Santa Maria della Misericordia University Hospital, 33100 Udine, Italy; delnegro.ilaria@gmail.com; 2 Neurology Unit, Head-Neck and Neurosciences Department, Santa Maria della Misericordia University Hospital, 33100 Udine, Italy; giada.pauletto@asufc.sanita.fvg.it (G.P.);; 3 Eye Clinic, Policlinico Umberto I, “Sapienza” University of Rome, 00142 Rome, Italy; 4 Department of Ophthalmology, Head-Neck and Neurosciences Department, Santa Maria della Misericordia University Hospital, 33100 Udine, Italy; 5 Neurosurgery Unit, Head-Neck and Neurosciences Department, Santa Maria della Misericordia University Hospital of Udine, 33100 Udine, Italy

**Keywords:** optic neuritis, genes, optic nerve, visual evoked potentials, retinal ganglion cells

## Abstract

Optic neuritis (ON) is an inflammatory condition affecting the optic nerve, leading to vision impairment and potential vision loss. This manuscript aims to provide a comprehensive review of the current understanding of ON, including its definition, epidemiology, physiology, genetics, molecular pathways, therapy, ongoing clinical studies, and future perspectives. ON is characterized by inflammation of the optic nerve, often resulting from an autoimmune response. Epidemiological studies have shown a higher incidence in females and an association with certain genetic factors. The physiology of ON involves an immune-mediated attack on the myelin sheath surrounding the optic nerve, leading to demyelination and subsequent impairment of nerve signal transmission. This inflammatory process involves various molecular pathways, including the activation of immune cells and the release of pro-inflammatory cytokines. Genetic factors play a significant role in the susceptibility to ON. Several genes involved in immune regulation and myelin maintenance have been implicated in the disease pathogenesis. Understanding the genetic basis can provide insights into disease mechanisms and potential therapeutic targets. Therapy for ON focuses on reducing inflammation and promoting nerve regeneration. Future perspectives involve personalized medicine approaches based on genetic profiling, regenerative therapies to repair damaged myelin, and the development of neuroprotective strategies. Advancements in understanding molecular pathways, genetics, and diagnostic tools offer new opportunities for targeted therapies and improved patient outcomes in the future.

## 1. Introduction

Optic neuritis (ON) is a condition characterized by the initial inflammation of the optic nerve, damage to the myelin sheath, and secondary injury to nerve fibers, leading to the apoptosis and death of retinal ganglion cells (RGCs), which further contributes to the ongoing vision impairment and potential vision loss. The cause of inflammation in ON often remains unknown. Nonetheless, it can be linked to different factors, including demyelinating lesions, autoimmune disorders, infectious agents, inflammatory conditions, and exogenous environmental factors. Recent studies have shown that alterations in the gut microbiome taxa have also been correlated with the risk of developing ON [1]. When all the causes of secondary ON have been excluded, the term idiopathic ON can be used. The prevalence of ON is variable between countries, but it is reported to be around 4–8 per 100,000 person years worldwide [2,3]. The etiology of inflammatory ON is multifactorial and involves autoimmune susceptibility, environmental factors, and genetic aspects.

Historically, ON has been classified as “typical”, usually subacute monolateral, and painless, with a good prognosis, and “atypical”, more often bilateral, with a high degree of visual acuity loss and worse prognosis [4]. Typical ON has been associated with multiple sclerosis (MS) and, in 20–42% of cases, is the presenting symptom of the condition [4,5,6]. In addition, almost half of patients with MS could manifest ON during any time of their disease span [7]. Atypical ON is usually associated with autoimmune disorders, out of MS, with particular regard to anti-aquaporin 4 (AQP4) antibodies disease or neuromyelitis optica spectrum disease (NMOSD) and anti-myelin oligodendrocyte glycoprotein (MOG) antibodies associated disease (MOGAD); these conditions are potentially much more vision-threatening and often lead to the necessity of long corticosteroid treatment or plasma exchange [8]. A new entity characterized by anti-glial fibrillary acidic protein (GFAP) antibodies has been recently identified in patients manifested with inflammatory CNS symptoms such as meningitis, encephalitis, and myelitis [9]. Visual involvement in GFAP astrocytopathy is characterized by asymptomatic bilateral optic disc edema and silent inflammation of the optic nerve up to severe bilateral vision impairment, whereas optic neuritis is rare [10,11].

Rheumatologic diseases can also manifest with atypical ON. In addition, bacterial and viral infections can cause atypical ON [12]. Rare cases of recurrent ON, without reaching the diagnostic criteria for MS, have been recently defined as chronic-relapsing inflammatory optic neuropathy (CRION). ON in CRION shows a good response to corticosteroid treatment in the acute phase, but needs to maintain prolonged immunosuppression in order to prevent relapses, which can be frequent and lead to RNFL damage and permanent vision loss [13].

Several articles provide a complete and accurate description of the clinical symptoms, etiologies, clinical course, and prognosis of known optic neuropathies/neuritis [10,14,15]. A 2022 position paper has provided a comprehensive overview of the diagnostic aspects and classification of ON [16]. The panel proposes a clinical definition of ON defined as a visual monocular, or rarer, binocular impairment with or without orbital pain worsening on eye movements, reduced contrast and color vision, and relative afferent pupillary deficit (RAPD). Supportive criteria can be derived from paraclinical examinations such as optical coherence tomography (OCT), magnetic resonance imaging (RMN), and humoral biomarkers [16].

Distinguishing between different forms of ON is crucial and can be quite challenging during the initial stages of the clinical progression.

Several studies have investigated the role of genetic polymorphisms of genes involved in immune mechanisms potentially related to the development of ON. Identifying alterations in gene expression in cell types within the optic nerve or immune cells involved in demyelination could potentially reveal novel therapeutic opportunities for addressing visual impairment in MS.

This review aims to provide a complete overview of the main genetic aspects and new insights in molecular analysis beyond the pathogenesis of ON and optic neuropathies.

## 2. Methodology

The authors developed inclusion criteria comprising published articles from 1995 (except for one original article from 1959) onward that were available in PubMed. Only full-length publications were considered for this research using the search string ‘‘optic neuritis” AND “genes” (58 results), ‘‘optic neuritis” AND “genetic” (182 results), or “hereditary optic neuropathy” AND “genes” (267 results) AND (1995:2023[pdat])”. Some articles were also derived from the bibliography of the chosen articles. Papers that were not in English, Italian, or Spanish language, outside the scope of this review, and duplicates, were excluded. A total of 507 records were returned in PubMed. A minimum of two of the authors reviewed each study for relevance.

## 3. The ON Model in Experimental Autoimmune Encephalomyelitis (EAE)

Inflammation of the optic nerve is a common feature in murine models of EAE. The infiltration of immune cells in the optic nerve begins 9 to 12 days after induction of EAE, and the demyelinating damage in the CNS is preceded by 1–2 days. It has been shown that global axonal loss of retinal ganglion cells (RGCs) is directly connected and secondary to nerve inflammation [17].

In a study conducted by Herold and colleagues on murine models of EAE, an augmented transcription of the amyloid precursor protein (APP) in RGCs in preclinical EAE (and not in the clinical phase of EAE) was found. The authors concluded that altered cleavage of APP in RGCs might lead to apoptotic loss of RGCs in the early stages of disease [18].

Astrocytes are one of the main cellular subtypes involved in inflammation and regulation of demyelination and, during inflammation, they manifest different gene expressions based on their anatomical region [19].

Astrocytic yes-associated protein (YAP) has been shown to play an anti-inflammatory role in a murine model of optic neuritis via activation of the transforming growth factor-β (TGF-β) pathway and was found to be upregulated after the induction of EAE and protect astrocytes by reducing optical nerve infiltration [20].

An RNA sequencing profile of the optic nerve astrocytes, to verify astrocyte-specific transcriptome changes during optic neuritis in EAE, showed an upregulation of genes involved in antigen presentation and complement activation and, on the other hand, a downregulation of cholesterol biosynthesis pathways, particularly 3-hydroxy-3-methylglutaryl-CoA synthase1 (*Hmgcs1*) and farnesyl-diphosphate farnesyltransferase 1 (*Fdft1*) genes. In particular, complement gene C3 significantly increased conferring to astrocytes a neurotoxic phenotype [19,21].

Cholesterols derived from astrocytes are transported to neurons and oligodendrocytes via ATP-binding cassette transporter (ABCA1) and apolipoprotein E (ApoE) [19]; thus, an alteration of any of these intermediate steps can lead to a dysfunction in myelin regeneration in CNS.

## 4. Idiopathic and MS-Related Typical ON

Momkute et al. identified APOE genotypes affecting the risk of developing ON. APOE is a protein involved in the transport and regulation of low-density lipoproteins (LDLs) and very-low-density lipoproteins (VLDLs). In their study, APOE serum levels were higher in the ON group than in the control group. Moreover, they found a correlation between the protein isoform coded by the gene APOE ε4/ε4 in male subjects and an increased risk of ON [22].

Punyte et al. investigated the role of three genes in the risk of developing ON in patients manifesting with isolated ON, MS, and healthy populations. Genotypes T/C and C/C of vascular endothelial growth factor A (VEGFA) rs1413711 were shown to be prevalent in the ON group. Furthermore an association between interleukin 6 (IL6) rs1800796 allele and the risk of ON in the MS group, compared to healthy subjects, was found [23]. Similar results were found by Gedvilaite et al. [24], which correlated allele C of VEGFA rs833068 with 1,7-fold increased odds of ON; moreover, they found a direct correlation.

Similarly, the IL6 rs1800795 G/G genotype and interleukin 1 receptor-like 1 (IL1RL1) rs1041973 genotype A/C have been associated with the risk of manifesting ON [24,25].

The receptor for advanced glycation end products (RAGE) is a multi-ligand receptor and a member of the immunoglobulin family that has been shown to be elevated in several neurological conditions, including Alzheimer’s disease, Parkinson’s disease, multiple sclerosis, myasthenia gravis, and cerebrovascular disease [26]. An Rs1800625 GG genotype was found to be associated with almost 7-fold increased odds of ON development, whereas the genotype AA seems to be protective [27].

The enzymes gelatinase A/matrix metalloproteinase-2 (MMP-2) and gelatinase B/MMP-9 promote neuroinflammation and neuroinflammatory processes and are essential for the induction of EAE. Moreover, MMP-9 has been demonstrated to be a key element for the infiltration of leukocytes into the central nervous system [28]. The MMP-2 rs243865 genotype C/T has been correlated to the risk of developing ON [6]. An inverse correlation of the G/A genotype of cholesteryl ester transfer protein (CETP) rs708272 was found with the risk of developing ON [24].

In addition, studies have been performed on human leukocyte antigen (HLA) expression and the risk of associated disease in patients with ON. In a 1993 study, the authors did not find any difference in HLA-DR15, -DR17, -DQA-1B, and -DQB-1B frequency between idiopathic ON and MS-associated ON [29]. Hauser et al. proved the association of HLA-DR2 in patients with ON (almost half of the patient group expressed this HLA) and the further diagnosis of definite MS [30]. HLA-Dw2 has been found to discriminate between patients with unilateral ON who manifested an MS-type ON (53%) and those with a reduced risk of MS who were HLA-Dw2-negative (47%) [31].

In recent years, large interest has grown in genetic studies and the correlations of gene mutations with clinical and prognostic factors, as well as in their correlation with pathogenic mechanisms.

A genome-wide association study (GWAS), performed in 2019 in MS patients, identified associations of different genes with visual system outcomes and indirectly linked to measures of disease severity and disability. In particular, the complement C3 gene was strongly associated with the degree of ganglion cell loss, while the C1QA rs158772 variant and CR1 rs61822967 variant were associated with an increased risk of visual impairment and decreased visual acuity [32].

A different study performed GWAS and quantitative RT-PCR analysis in the peripheral blood of patients with ON and the exclusion of secondary autoimmune/infective disease. The group found 722 differently expressed genes in patients with ON with respect to healthy controls. Of these, 377 exhibited an increased expression and 345 exhibited a decreased, expression. Moreover, differential expression of eight genes was demonstrated in the ON group, with *SLPI* (secretory leukocyte peptidase inhibitor), *CR3* (complement receptor 3), and *ITGA4* (integrin subunit α 4) being the most significantly represented. The main differences in gene expressions involved functions regarding protein phosphorylation, apoptosis inhibition, cell cycles, T and B cell functions, and anti-inflammatory CNS pathways, leading to an increase in inflammatory processes in the CNS [33].

These technological advancements highlight the powerful role of gene analysis in the diagnostic process and the correct assessment of the predisposition of some patients with MS or ON to develop a more rapid retinal neurodegeneration or visual function impairment.

## 5. Atypical ON

### 5.1. Autoimmune ON

An RNA sequencing analysis, performed in blood samples of patients affected by AQP4-ON and MOG-ON, showed differences in gene expression in these two entities. In AQP4-ON, inflammatory pathways involve toll-like receptors (TLR)-2, TLR5, TLR8, and TLR10, and the main mechanism of inflammations is conducted by a damage-associated molecular pattern (DAMP); MOG-ON patients manifest a major transcription of TLR1, TLR2, TLR4, TLR5, and TLR8, and inflammation is mediated primarily by a pathogen-associated molecular pattern (PAMP) [34]. Genetic aspects in the susceptibility of atypical ON have been recently demonstrated in Mexican populations [35]. In this study, the authors found that the class II HLA alleles were associated with NMO development (HLA-DQB1*03:01, -DRB1*08:02, -DRB1*16:02, -DRB1*14:06, and -DQB1*04:02) in individuals belong to a Native American ancestry (instead of European lineage, which is also diffuse in Latin American populations). A recent whole-genome sequence study demonstrated new genetic risk factors for NMO. The authors found associations with HLA-DRB1*03:01 (a known risk factor for NMO) and HLA-DRB1:15:01, which is a well-known risk factor for MS [36]. In addition, a reduced copy number in the region coding for complement component 4 (C4) was noted in seropositive NMO-IgG+ patients, who mostly carry only a single short C4B gene. Furthermore, NMO-IgG+ patients showed reduced C4a protein serum levels compared with the controls. The authors suggested a common role of these findings between seropositive NMO and systemic lupus erythematosus and stated that genetic risk factors for NMO-IgG+ are more closely linked to those of SLE [36]. Differences in gene expression and gene polymorphisms are also involved in the treatment response, especially in an acute setting. An ATP-binding cassette superfamily B member 1 (ABCB1) G2677T/A (rs2032582) polymorphism was associated with glucocorticoid response in AQP4-IgG+ NMOSD-ON patients [37]. An ABCB1 gene codifies glycoprotein P, which is involved in the transport of endogenous and exogenous substances from the CNS to the blood, thus maintaining the correct homeostasis [38] and cholesterols and lipids, as previously cited. Patients manifested with the GG genotype, instead of the T/A T/A genotype, have a significantly higher risk of developing corticosteroid resistance. Changes in visual acuity scores in patients with the GG genotype are significantly lower than patients with the T/A T/A genotype. Moreover, the corticosteroid-sensitive group manifested a lower frequency of the GG genotype. Thus, G2677T/A GG and G T/A genotypes have been shown to increase the corticosteroid resistance risk up to 3,5 times in NMOSD-ON patients [37].

### 5.2. Genetic ON and Mitochondrial DNA (mtDNA) Mutations

Several mtDNA mutations are recognized in the setting of diseases that lead to atrophic damage of the optic nerve.

Leber hereditary optic neuropathy (LHON) is a rare maternally hereditary disease that typically presents with bilateral, cecocentral, painless, and subacute visual impairment and usually becomes permanent after years [15]. Historically, point mutations in mtDNA were associated with disease onset, and three primary recurrent mutations account for almost the whole genetic susceptibility (90% of all cases) [39].

The primary mutations, identified by bi-directional fluorescent Sanger sequencing are, in order of frequency, m.11778G > A, m.3460G > A, and m.14484T > C, with the latter being the only one associated with an increased possibility of visual improvement after the acute phase [15]. This mutation involves genes of the complex I of the mitochondrial respiratory chain. In almost 10–15% of cases, these mutations are heteroplasmic, a blend of both mutated and normal mtDNA [39], underlying the high variability of phenotype expression and the incomplete penetrance of this disease. There is a variable risk of lifetime visual loss in people carrying homoplasmic primary mtDNA variants per LHON (from a minimum of 9% in females to a maximum of 51% in males) [40].

Moreover, it has been suggested that primary mutations themselves are insufficient to justify all the clinical phenotypes and that other environmental and/or genetic factors should contribute to the phenotypic expression and penetrance of primary mtDNA mutations. The male sex, age (later age of onset is related to a lower chance of visual loss), and smoking (associated with an increased risk of vision loss) are believed to be the most extra-genetic factors involved in the phenotype expression of this disease [40,41].

In this setting, numerous different mutations in mtDNA are arising. If the main primary mutations are not detected, a mitochondrial multigene panel for genes, known to be involved in the disease, or complete mtDNA sequencing should be performed [40].

There are many reports in the literature that analyze the whole mitochondrial gene expression in LHON in different familiar clusters, showing distinct sets of mtDNA polymorphism belonging to different haplogroups, and reporting unknown variants. However, the real impact of secondary mutations on disease onset and progression remains to be clarified.

The most common inherited optic neuropathy is Dominant Optic Atrophy (DOA) or Kjer’s Optic Atrophy, first described in 1959 [42] and has an estimated frequency of 1/30,000–50,000 cases [43,44]. DOA is an autosomal disease in which the altered nuclear chromosomal gene leads to mitochondrial dysfunction, which especially impacts RGCs [45], thus conferring the phenotype of mitochondriopathy [44]. With time and the discovery of multiple gene and loci variants of this disease, the clinical spectrum of DOA has largely expanded. Clinical characteristics of the disease are not discussed in this article; however, the spectrum of the disease comprises isolated bilateral optic neuropathy and DOAplus variants, which represent almost 20% of all patients, manifest extra-ophthalmologically, and are usually more severe [46,47]. The most common extraocular symptom in DOA+ variants is deafness, followed by external ophthalmoplegia, myopathy, ataxia, and neuropathy [47].

OPA1, located in chromosome 3, is the first and most common gene involved in the pathogenesis of DOA, and is usually inherited in an autosomal dominant manner. Disease phenotypes and penetrance are fairly heterogeneous with asymptomatic carriers of pathogenic variants [48]. OPA1 is a protein of the inner mitochondrial membrane and is involved in multiple cellular functions, such as fission and fusion of mitochondria, oxidative phosphorylation, calcium homeostasis, and mitochondrial homeostasis, which are mandatory in order to adapt the activity of mitochondria to current energetic demands [49,50].

To date, more than 500 variants of the OPA gene have been described, of which more than 80% are considered pathogenic [51], most of them leading to an alteration of the GTPase domain or dynamic domain of the protein [51]. Other OPA loci have been identified in several other chromosomes, including the X chromosome, and some of them are inhered in a recessive or X-linked mode.

DOA+ phenotypes more often manifest missense mutation or mutations of the GTPase region [47]. A novel recurrent missense variant has been identified as a strong contributor to the DOA+ syndrome (c.1499G > a mutation in the GTPase domain) [51].

Optic atrophy has also been reported in patients with other multisystemic mitochondrial diseases. Clinical manifestations of DOA+ patients are often also characteristic of mitochondrial diseases, thus creating a more complex diagnostic scenario. Some of these syndromes are maternally inherited Leigh syndrome (MILS), myoclonic epilepsy associated with ragged-red fibers (MERRFs), the syndrome of neuropathy, ataxia, retinitis pigmentosa (NARP), mitochondrial myopathy, encephalopathy, lactic acidosis and stroke-like episodes (MELAS), mitochondrial neuro-gastrointestinal encephalopathy (MNGIE), chronic progressive external ophthalmoplegia (CPEO), and the Kearns–Sayre syndrome (KSS) [39].

Other autosomal and non-mitochondrial rare diseases often enter the differential diagnosis of genetic optic neuropathies and present similar and overlapping characteristics.

Autosomal recessive mutations of the Wolfram syndrome (WFS1) gene and, less frequently, the CDGSH iron–sulphur domain-containing protein 2 (CIDS2) gene located in chromosome 4, cause a disease called Wolfram syndrome (WS), or DIDMOAD, which is characterized by juvenile-onset diabetes, progressive optic atrophy, diabetes insipidus, sensorineural hearing, urinary tract problems loss as well as other neurological symptoms, brain stem atrophy, and psychiatric comorbidities [52,53]. WF genes are involved in the maintenance of the correct homeostasis of the endoplasmic reticulum [54]. More than 170 different mutations of the WFS1 gene have been found, justifying different phenotypes of the disease [55,56].

A comprehensive list of nuclear autosomal genes involved in diseases that cause isolated or syndromic optic atrophy has recently been proposed by Newman and colleagues [15].

Numerous studies have analyzed the role of mtDNA mutations in ON and the risk of developing MS. One of the first studies conducted in patients with MS with and without ON and healthy controls demonstrated a correlation between the mitochondrial haplogroup J and ON. Haplogroup J is characterized by two mutations at the nucleotide positions 4216 and 13,708, while an isolated mutation of position 13,708 is identified as haplogroup X. This feature is normally present in the general population (and so no difference was found in prevalence between the MS and the control group); however, only the patient who manifested ON carried haplogroup J, which, on the contrary was absent in patients without ON [57]. This finding supported the concept that mutations in mtDNA could link optic hereditary neuropathy and MS in a multifactorial way.

Another study did not find a different distribution of mtDNA mutations between patients suffering from MS and the controls; however, there was an almost significant difference in the presence of haplogroup J and X in patients who manifested ON, which was present in all but one patient in whom ON was the initial symptom [58].

Mitochondrial dysfunction and systemic mitochondrial abnormalities are well-known mechanisms of hereditary optic neuropathies; however, an increasing amount of evidence suggests their roles in neurodegenerative disease, even in the degeneration of RGCs and optic nerve axons. In addition, carriers of LHON mutations often manifest optic neuropathies and MS-like disorders and are believed to be a risk factor for optic nerve inflammation [59,60].

Several groups have studied the impact of anti-oxidative therapies (such as NADH-dehydrogenase type-2, mitochondrial heat shock protein 70) that act on the mitochondrial respiratory chain by preventing or ameliorating retinal thinning in EAE and optic nerve axons apoptosis [61,62].

## 6. Future Perspective and Treatments

### 6.1. Experimental Neuroprotective Therapies

Myelin preservation and the regeneration of oligodendrocytes are the greatest issues in the research of potential optic nerve therapies. Numerous studies, mostly on animal models, have been conducted in this contest.

The activation of T cells needs several steps, such as the binding of T cell receptors (TCRs) and CD4 to the complex of the major histocompatibility complex (MHC) class II/peptide on antigen-presenting cells and the binding of costimulatory receptors and ligands. The overstimulation of T cells with an induced TCR pathway and without the presence of co-stimulation leads to a state of anergy. In this way, artificial stimulation of antigen-specific CD4+ T cells by TCR ligation in the absence of co-stimulation stimuli induces immune tolerance and reduces disease activity [63]. Previous studies on recombinant T cell receptor ligands (RTLs) were conducted in MS patients, suggesting a potential role for this treatment in limiting inflammation and promoting remyelination [64].

In a study conducted by Adamus and colleagues, the effect of RTL immunotherapy on the development of ON was explored in a murine model of humanized HLA-DR3 mice. After the induction of EAE and the development of optic nerve inflammation (which appears 8–9 days before the symptoms of EAE), the administration of RTL stops the migration of inflammatory cells to ON, protects the long-term demyelination of ON, and reduces RGC apoptosis [65].

Calpain is a calcium-dependent proteolytic enzyme that destructs myelin proteins and other cytoskeletal substrates (for example, myelin basic protein, neurofilament protein, and spectrin) and is thus responsible for both demyelination and neuronal cell death. As calpain activity increased in EAE, target therapies with antagonists of calpain have been shown to promote neuroprotective pathways, reduce inflammation, downregulate the expression of proapoptotic proteins and pro-inflammatory molecules and activated immune cells, and prevent apoptosis of oligodendrocytes and demyelination [66,67].

Matsuda et al. proposed the transfection of Calcitonin gene-related peptide (CGRP)-expressing dendritic cells in mice after the induction of EAE and demonstrated a prominent anti-inflammatory role for this peptide in the modulation of inflammation (increasing the expression of interleukin 10 by spleen cells and the augmented expansion of CD4 + CD25 + FoxP3 + regulatory T cells) and the reduction in ON [68]. The same group, in a similar way, also tested the transfection of interleukin 10-expressing dendritic cells. In the transfected group, a reduction in pro-inflammatory cytokines secretion and an augmented IL10 production in lymph nodes were shown, as well as an accumulation of regulatory T cells in the spleen and lymph nodes. In addition, a suppression of optic nerve infiltrating cells, an amelioration of EAE symptoms, and reduced ON frequency have been reported [69].

A more recent target of neuroprotective agents is the sterile α and TIR motif-containing protein 1 (SARM1). This protein is a nicotinamide adenine dinucleotide (NAD) hydrolase responsible for the degradation of axonal NAD and the subsequent activation of the Wallerian-like degeneration pathway derived from axonal damage [70]. A recent study showed a beneficial effect of SARM1 inhibition (both via antisense oligonucleotide and gene modulation/knockout) in traumatic and hypertensive damage of the optic nerve but not demyelinating ON [71].

### 6.2. Experimental Anti-Oxidant Gene Therapy

Anti-oxidant therapies were studied in the early reports of the experimental models.

The human catalase gene was inserted into a recombinant adeno-associated virus and intravitreally administrated into the eye of transgenic mice; treated mice manifested a reduced demyelination and cellule infiltration of the optic nerve and an amelioration of the blood–brain barrier integrity [72], and these results were even more pronounced when administration was performed in transgenic mice overexpressing extracellular superoxide dismutase (ECSOD) in dual gene therapy [73]. The combination therapy with ECSOD and catalase recombinant adenovirus provided long-term protection against RGC loss, optic nerve demyelination, and cellular infiltration [74].

The augmented expression of silent information regulator 1 (sirtuin-1/SIRT1) and nuclear factor (erythroid-derived 2)-like 2 (NRF2) via a recombinant adeno-associated virus vector has been shown to reduce RGC apoptosis and ameliorate visual loss in mice with EAE [75,76]. SIRT1 is a ubiquitous NAD+-dependent deacetylase involved in the prevention of oxidative stress and inflammation and is thus involved in the anti-oxidant cellular processes [76]. NRF2 is a member of the subfamily of basic region leucine zipper transcription factors and is involved in the cellular resistance to oxidative stress [77].

### 6.3. Experimental Optic Nerve Regenerative Therapies

RGCs are neurons that are not able to regenerate, and their damage causes sustained visual loss [78]. Optic nerve regenerative therapies are a major challenge in neurodegenerative and inflammatory optic nerve disease. Some studies explored the role of mesenchymal stem cell (MSC) transplantation in degenerative ocular disease and optic neuritis. MSCs are multipotent cells able to differentiate into various cellular types. They are not only involved in the restoration of damaged cells but they are also thought to promote paracrine effects both trophic and immunomodulatory (angiogenic, anti-apoptotic, and supportive pathways) and stimulate the proliferation of precursor cells and cell growth and survival [79].

Intravitreal MSC injections have been shown to reduce in vivo intraocular pressure artificially induced in murine models of glaucoma and, in vivo, to promote anti-apoptotic pathways and decrease profibrotic events [80]. Furthermore, MSCs can support the mitochondrial function of RGCs, protecting them from oxidative damage [81]; in addition, intravitreal injection of neuronal steam cell-based factors confers long-lasting protection against the axotomy-induced cell death of RGCs in mice [82,83]. A similar group of stem cell-derived neuronal progenitor cells has also demonstrated a protective role on optic nerve damage by upregulating neuroprotective factors to enhance anti-inflammatory proteins in murine models [84].

### 6.4. Experimental Therapies in Human Model Optic Neuropathies

Even if mice model data are promising, only a few data points on patients with ON or MS-ON are available.

A phase 2 clinical trial was performed in 2012, with autologous mesenchymal stem cells parenteral infusion in patients with progressive forms of MS. Treated patients manifested visual acuity improvement, a reduction in visual evoked potential latency, and an increased optic nerve area; however, no effects on RNFL thickness were recognized [85]. Similarly, a study conducted on 15 patients with NMOSD who underwent a single intravenous infusion of MSCs showed the capacity of MSCs to increase RNFL thickness and optic nerve diameters [86].

Promising treatment with MSCs is, however, burdened by both technical and conceptual limitations, such as the risk of malignant transformation and the risk of anterograde or retrograde degeneration, which can sustain visual impairment even in regenerated optic nerve [87]; fortunately, new treatment strategies are being explored in the field of regenerative therapies, with MSCs-derived extracellular vesicles being one of the most promising. An extensive review of this topic has been performed by Aneesh and colleagues [88].

Furthermore, new insights are emerging in the modulation of oligodendrocyte differentiation and myelination. The modulation of intra- and extracellular pathways involved in the terminal differentiation of optic nerve oligodendrocytes will be a promising therapy [89].

In the setting of hereditary genetic disorders of the optic nerve, gene therapy has been recently investigated. Anti-oxidative therapies have been tried and largely used in LHON in order to ameliorate altered mitochondrial respiration with scarce results [90]. The only therapy approved for LHON in Europe is idebenone, a quinone analog, but its benefit depends largely on the type of mutation; having the m.11778G>A mutation is the worst prognosis [91].

Several trials have been recently performed for patients carrying the m.11778G>A mutation with a new technology of gene replacement mediated by an adeno-associated virus. Intravitreal administration of this gene therapy (named lenadogene nolparvovec) is supposed to supplement the defective gene, leading to an improvement in visual acuity after vision loss, especially when bilaterally administered [92,93,94].

## 7. Conclusions

Greater access to molecular genetic testing has expanded the phenotype associated with specific disease-causing genes, and we have used a broad classification based on whether the patient exhibits isolated or syndromic optic atrophy.

The possibility of identifying early genetic alterations could lead, primarily, to an earlier and precise classification of the disease and, secondarily, to a better prognostic framework and a more rapid and accurate addressing of the patients. The vast, and always richer, panel of genetic alterations of optic neuropathies should be taken into consideration in the diagnostic algorithm. Advanced and more rapid genetic tests are available, thereby contributing to improved genetic counseling and the family members’ awareness of the inheritance pattern of the disease.

Treating typical ON primarily accelerates recovery, without any effect on the final visual outcome. However, the diagnosis of ON or CIS often requires close clinical monitoring and further delay in the early initiation of disease-modifying therapies. The possibility to access rapid genetic tests could be a great improvement in this setting.

Several studies reported in this review found an association, both in murine and human models, between recurrent pathways, especially those involved in the cholesterol synthesis pathway, and genes involved in the complement activation that seem to play a central role in the pathogenesis of optic disease and an increased risk of optic nerve damage or predisposition to oxidative injury.

Finally, this review highlights the importance of testing genetics in patients presented with optic atrophy associated with other neurological conditions, which could mislead the clinician to other disease diagnoses, with particular attention to spastic paraplegia, multiple sclerosis-like illness, and ataxia. The detection of gene alterations is not only important for understanding and recognizing the underlying disease but also improving experimental research, as gene therapy represents a promising therapeutic approach for otherwise untreatable pathologies. A summary of the main genes involved in the pathogenesis of non-hereditary optic neuritis is reported in Table 1.

## Figures and Tables

**Table 1:** Altered genes involved in the pathogenesis of non-hereditary ON.

Type on Inflammatory ON	Altered Gene	Hypothesized Mechanism
Murine model of EAE	Amyloid precursor protein (APP)	Apoptosis of RGCs
	Yes-associated protein (YAP)	Reduction in optic nerve infiltration
	3-hydroxy-3-methylglutaryl-CoA synthase1 (*Hmgcs1*)	Downregulation of cholesterol biosynthesis in astrocytes
	Farnesyl-diphosphate farnesyltransferase 1 (*Fdft1*)	Downregulation of cholesterol biosynthesis in astrocytes
	Complement C3	Neurotoxic astrocytes
Idiopathic/MS-related typical ON	Apolipoprotein E (APOE)	Altered transport of fat molecules
	Vascular endothelial growth factor A (VEGFA)	Unknown (vascular?)
	Interleukin 6 (IL6)	Pro-inflammatory environment
	Interleukin 1 receptor-like 1	Pro-inflammatory environment
	Receptor for advanced glycation end products (RAGE)	Activation and persistence of inflammation
	Gelatinase A/matrix metalloproteinase-2 (MMP-2) and gelatinase B/MMP-9	Apoptosis of RGCs
	Cholesteryl ester transfer protein (CETP)	Altered transport of fat molecules
	Complement C3, C1QA, and CR1	Damage to the optic nerve
	SLPI, CR3, and ITGA4	Pro-inflammatory environment
Atypical ON	Toll-like receptors (TLRs)	Activation of pro-inflammatory pathways
	ATP-binding cassette superfamily B member 1 (ABCB1)	Altered transport of fat molecules

## Data Availability

Not applicable.
